# Algorithmic design of a noise-resistant and efficient closed-loop deep brain stimulation system: A computational approach

**DOI:** 10.1371/journal.pone.0171458

**Published:** 2017-02-21

**Authors:** Sofia D. Karamintziou, Ana Luísa Custódio, Brigitte Piallat, Mircea Polosan, Stéphan Chabardès, Pantelis G. Stathis, George A. Tagaris, Damianos E. Sakas, Georgia E. Polychronaki, George L. Tsirogiannis, Olivier David, Konstantina S. Nikita

**Affiliations:** 1 School of Electrical and Computer Engineering, National Technical University of Athens, Athens, Greece; 2 Department of Mechanical Engineering, University of California, Riverside, California, United States of America; 3 Department of Mathematics, FCT-UNL-CMA, Caparica, Portugal; 4 Univ. Grenoble Alpes, Grenoble Institut des Neurosciences, GIN, Grenoble, France; 5 Inserm, U1216, Grenoble, France; 6 Department of Psychiatry, University Hospital of Grenoble, Grenoble, France; 7 Department of Neurosurgery, University Hospital of Grenoble, Grenoble, France; 8 Department of Neurology, Mediterraneo Hospital, Athens, Greece; 9 Department of Neurology, ‘G. Gennimatas’ General Hospital of Athens, Athens, Greece; 10 Department of Neurosurgery, University of Athens Medical School, ‘Evangelismos’ General Hospital, Athens, Greece; University of California Los Angeles, UNITED STATES

## Abstract

Advances in the field of closed-loop neuromodulation call for analysis and modeling approaches capable of confronting challenges related to the complex neuronal response to stimulation and the presence of strong internal and measurement noise in neural recordings. Here we elaborate on the algorithmic aspects of a noise-resistant closed-loop subthalamic nucleus deep brain stimulation system for advanced Parkinson’s disease and treatment-refractory obsessive-compulsive disorder, ensuring remarkable performance in terms of both efficiency and selectivity of stimulation, as well as in terms of computational speed. First, we propose an efficient method drawn from dynamical systems theory, for the reliable assessment of significant nonlinear coupling between beta and high-frequency subthalamic neuronal activity, as a biomarker for feedback control. Further, we present a model-based strategy through which optimal parameters of stimulation for minimum energy desynchronizing control of neuronal activity are being identified. The strategy integrates stochastic modeling and derivative-free optimization of neural dynamics based on quadratic modeling. On the basis of numerical simulations, we demonstrate the potential of the presented modeling approach to identify, at a relatively low computational cost, stimulation settings potentially associated with a significantly higher degree of efficiency and selectivity compared with stimulation settings determined post-operatively. Our data reinforce the hypothesis that model-based control strategies are crucial for the design of novel stimulation protocols at the backstage of clinical applications.

## Introduction

The use of electrical deep brain stimulation (DBS), during approximately the last 30 years, has been proven to provide striking benefits for patients with advanced Parkinson’s disease (PD), essential tremor and dystonia [1–4] who have failed conventional therapies. In the interim, promising applications of this technique for the treatment of neuropsychiatric disorders have emerged, including treatment-refractory obsessive-compulsive disorder (OCD), Tourette’s syndrome, major depressive disorder, drug addiction and anorexia nervosa [5–9]. Challenges however exist and are principally related to the optimization of the efficiency of stimulation through refined strategies at multiple peri-operative levels. Particularly, in addition to the appropriate patient selection and anatomical target determination [10], the outcome of DBS may be strongly influenced by the quality of post-operative clinical management, i.e., the adjustment of stimulation parameters and the selection of the optimal contact, usually over periods of weeks to months [11]. Apart from being considerably time consuming, this trial-and-error procedure may not necessarily yield the optimal trade-off between maximal therapeutic benefit and minimal stimulation-induced side-effects [12]. Moreover, it fails to keep pace with the fact that movement and neuropsychiatric disorder symptoms may fluctuate over significantly shorter time-scales of seconds to days. Chronically, the open-loop nature and monomorph pattern of conventional high-frequency stimulation appears to favor tolerance/habituation phenomena, while being associated with a significant rate of power consumption [13].

Against this background, closed-loop DBS is emerging as a more robust alternative and one of the most promising breakthroughs in the field of neuromodulation [14, 15]. In an optimal closed-loop-stimulation scenario, delivery of maximally efficient *stimulation protocols* is adjusted to the fast dynamics of movement and neuropsychiatric disorder symptoms through utilization of specific *biomarkers* that capture the patient’s clinical state in real time [16]. Hence, any algorithm designed for a maximally efficient closed- loop DBS system shall conceptually satisfy two core specifications [17]: the *reliable* assessment of optimal neurophysiological biomarkers for feedback control and the *robust* identification of alternative stimulation protocols that may be more therapeutically- and energy-efficient compared with the conventional pattern of stimulation [18, 19].

*Nonlinear coupling* across multiple frequency bands in the basal ganglia and in cortical structures is being increasingly highlighted as a potentially predictive biomarker of PD and OCD pathophysiology [20–25]. To date, assessment of this biomarker has largely relied on evaluation of phase-amplitude coupling by means of the *Hilbert transform* combined with linear band-pass filtering [20, 26]. Remarkably however, the respective phase reconstruction method may be characterized by a high level of susceptibility to *measurement noise* and a high rate of artificial *phase slips* [27, 28], thereby discarding possibly rich information that would be revealed by employing noise-resistant or phase reconstruction-free methods [29, 30]. Meanwhile, *model-based control* policies for the determination of temporally alternative stimulation protocols [31–41], though still limited, are most commonly oriented towards the minimum energy *desynchronizing control* of neuronal activity. The rationale behind this objective lies in indications that temporally alternative DBS waveforms, including *stochastic* waveforms, hold the potential to drive the neuronal dynamics within the basal ganglia back to the *normal desynchronized state*—namely to more irregular and less burst-like firing patterns [19, 42]—thereby outperforming the action of standard DBS waveforms, the mechanism of which has been principally attributed to the reinforcement-driven regularization of neural firing patterns in the vicinity of the stimulated nucleus [43–45].

**Fig 1:**
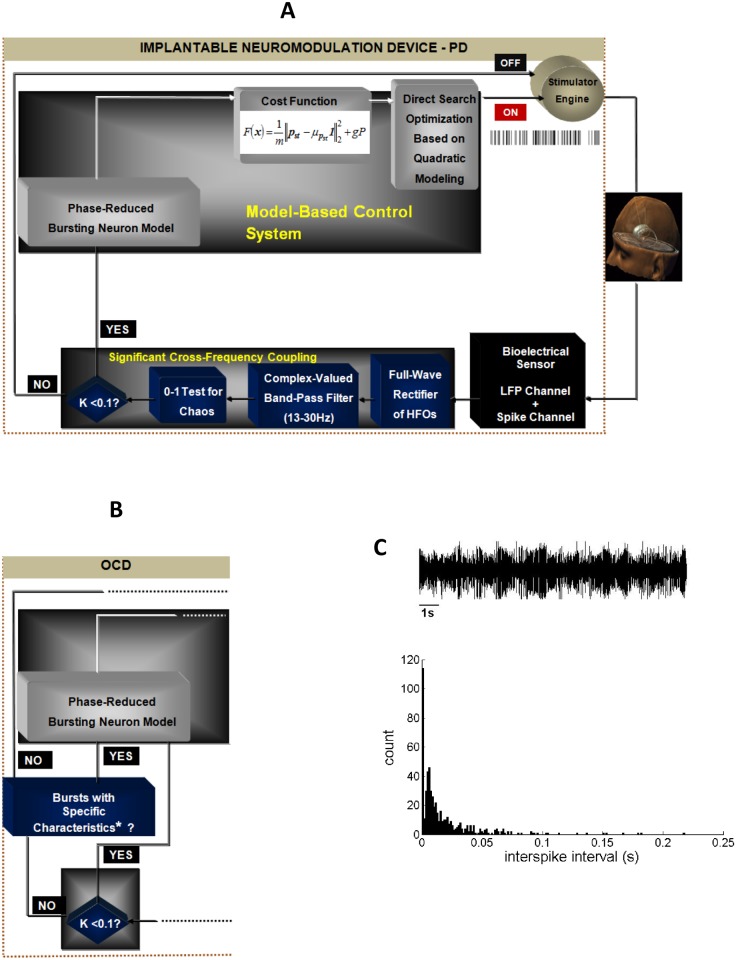
Schematic of the closed-loop DBS system. (A) Case of advanced PD; LFP: local field potential; HFOs: high-frequency oscillations; head model reproduced with permission from Inserm /Eric Bardinet, Jerome Yelnik and Luc Mallet. (B) Case of treatment-refractory OCD. Applicability of cross-frequency coupling as a biomarker for feedback control in case of treatment-refractory OCD may be subject-specific. Accordingly, the presence of bursting neuronal activity was included as an alternative biomarker for feedback control in the respective closed-loop neuromodulation scheme; *short interburst interval and high intraburst frequency. (C) Exemplary identification of a bursting firing pattern of neuronal activity at Central, -2.56mm, Left STN, case O2 (top) based on the interspike interval (ISI) histogram (bottom). The histogram is characterized by a positively skewed distribution indicating a large fraction of short ISIs and a high intraburst frequency (*μ*_ISI_ = 0.0179s; *Var*_ISI_ = 0.00069).

Considering these observations collectively and employing concepts drawn from dynamical systems and control theory, in this study we elaborate on the algorithmic aspects of a noise-resistant and efficient closed-loop neuromodulation system for advanced PD and treatment-refractory OCD (Fig 1A and 1B). Specifically, we first state a series of methods robust to the presence of internal and measurement noise that are employed in order to reliably assess significant nonlinear coupling between beta and high-frequency subthalamic activity, as a biomarker for feedback control in the closed-loop neuromodulation scheme. We then suggest a model-based control strategy through which optimal parameters of stimulation for minimum energy desynchronization of neuronal activity are being identified.

In our analysis, we opt for a phase-reduced bursting neuron model [40, 46, 47]. Our motivation for this particular selection is twofold. First, *phase reduction* theory constitutes a powerful mathematical framework for the analysis of the synchronization and response properties of *nonlinear* oscillatory activity based on a single *phase* variable [48]. Second, since *bursting* activity is a prominent characteristic of subthalamic neuronal activity in PD and OCD (Fig 1C) [49–52], a qualitative model of neuronal bursting, like the well-established Hindmarsh-Rose model for bursting, may be a highly appropriate point of reference for capturing the respective neuronal dynamics [31, 53]. It should be noted that, depending on parameter selection, the Hindmarsh-Rose model may capture a wide range of neuronal dynamics: from regular spiking to bursting to chaotic regimes and fixed-point behavior. However, in this study, we focus on a computational model able to qualitatively capture pathological neuronal dynamics, i.e., bursting behavior. Accordingly, a major part of the phase-response dynamics of the reduced model has been determined based on the Hindmarsh-Rose model for bursting [46, 47]. Importantly, the employed phase-reduced model, which simulates the effect of stimulation on pathological neuronal activity, is *data-driven*, i.e., microelectrode recordings (MERs) acquired during subthalamic nucleus (STN) DBS surgical interventions for PD and OCD are used to estimate the unknown model parameters off-line. Thereby, the ability of the model to simulate realistic neuronal dynamics is further enhanced. A data-driven phase-reduced model of subthalamic neuronal activity was employed in [40], where we adopted a measure of the *invariant density* (steady-state phase distribution) of the simulated dynamical system, as a quantity inversely related to the *desynchronizing effect* of temporally alternative patterns of stimulation, and further provided evidence for a possible correlation of this measure with *clinical effectiveness* of stimulation in PD. Determination of the precise optimal parameters of stimulation is accomplished through the application of a *derivative-free optimization* algorithm, in particular a model-based pattern search method guided by simplex derivatives [54, 55]. This approach is motivated by the fact that the neural response to DBS parameters is expected to be a complex, *non-differentiable* function [19, 56].

Finally, extending the results of our previous work, we attempt to provide indications for a possible correlation of the invariant density measure with clinical effectiveness of stimulation in treatment-refractory OCD. Overall, the results of this study corroborate the ability of the presented modeling approach to identify stimulation settings potentially associated with a significantly higher degree of *efficiency* and *selectivity* compared with stimulation settings determined post-operatively, while guaranteeing a relatively low computational cost.

## Materials and methods

### Data description

We used MER data acquired during 8 STN-DBS surgical interventions for advanced PD at Evangelismos General Hospital of Athens and 8 STN-DBS surgical interventions for treatment-refractory OCD at Grenoble University Hospital. Surgery was performed after provision of written informed consent by patients with advanced PD and after consideration of strict ethical guidelines and inclusion criteria for patients with treatment-refractory OCD (the consent procedure was approved by the National Consultative Ethics Committee on Health and Life Sciences 2002). Patients’ clinical characteristics have been described in detail elsewhere (cases 1–5 and 7–9 [28]; cases O1-O6 and O9 [49]; case P5 [57]). In case of PD, medication was withdrawn at least 12 hours before surgery. All recordings were obtained, while patients were awake and at rest, using five tungsten microelectrodes (2mm apart, tip diameter < 25 *μ*m, Medtronic Inc., Minneapolis, MN (Evangelismos General Hospital of Athens) / FHC Inc., Bowdoinham, ME, USA (Grenoble University Hospital)). Signals acquired during STN-DBS for advanced PD were pre-amplified, band-pass filtered between 0.1 Hz and 10 kHz, sampled at 24 kHz (Leadpoint TM Neural Activity Monitoring System, Medtronic Inc., Minneapolis, MN) and off-line band-pass filtered at 1-300Hz and 300Hz-6 kHz, applying four-pole Butterworth filters (Matlab, Mathworks, Natick, MA). Signals acquired during STN-DBS for treatment-refractory OCD were pre-amplified, band-pass filtered at two frequency bands (1–300 Hz and 300 Hz-6 kHz) and sampled at 3 kHz and 48 kHz, respectively (Neurotrek System, Alpha-Omega Engineering, Nazareth, Israel). A total of 31 acceptable MER trajectories (i.e., trajectories traversing the broadest extent of the nucleus) acquired during STN-DBS for PD and 12 acceptable MER trajectories acquired during STN-DBS for OCD were selected for off-line analysis. Preprocessing of each MER included its subdivision into three distinct neuronal populations: spiking activity acquired by employing a five-point spike template [28, 58], background unit activity reconstructed according to [59], and local field potential (LFP) activity (1-300Hz). In order to keep pace with the employed phase reduced bursting neuron model, following the assessment of the biomarker for feedback control, optimal parameters of stimulation were determined only for sites at which bursting activity was recorded. A bursting or burst-like firing pattern of neuronal activity was identified according to [60] (Fig 1C).

### Noise-resistant assessment of cross-frequency coupling as a biomarker for feedback control

The first objective of this study was to propose a method for the assessment of nonlinear coupling between beta and high-frequency activity characterized by reduced sensitivity to internal and measurement noise compared with the combined application of linear band-pass filtering and the Hilbert transform [20–22, 24, 26]. We therefore employed a two-part technique for the designed scheme (Fig 1). First, the beta-band-frequency (13-30Hz) envelope of the high-pass filtered (200-300Hz) LFPs (or, alternatively, of the high-frequency signal component (300-500Hz)) was assessed. Particularly, the high-pass filtered signal was full-wave rectified, mean subtracted and downsampled to 1kHz. The derived signal was band-pass filtered at 13-30Hz by applying the *complex-valued filter* proposed by [29]. This filter is designed based upon minimization of the relative variance

$$q^{2}=\frac{\text{var}{\left|z\right|}^{2}}{{\langle {\left|z\right|}^{2}\rangle }^{2}}=\frac{\langle {\left|z\right|}^{4}\rangle }{{\langle {\left|z\right|}^{2}\rangle }^{2}}-1,$$ (1)

where *z* is the filter output. In particular, z = f*x, where *f* is the filter’s impulse response, *x* is the given signal and ‘‘*” denotes convolution. The robustness of this filter lies at its property to increase the signal to measurement noise ratio with respect to the complete dynamics of its impulse response. Moreover, this filter has been proven to cope with strong internal noise that constitutes a prominent characteristic of the recorded subthalamic neuronal activity. Accordingly, the presented method accounts not only for the presence of measurement noise, but also for the presence of intrinsic noise which is relevant to PD and OCD pathophysiology. Following the employment of the complex-valued filter, we applied the *0–1 test for chaos* [30] to a logarithmic transformation of the complex magnitude of the filter output in order to assess the presence of significant nonlinear coupling between beta and high-frequency activity in the STN of patients with PD or OCD. Nonlinear coupling corresponds to regular, non-chaotic dynamics indicated by a test outcome approximately equal to 0.

In addition to being a phase reconstruction-free method for the determination of regular or chaotic dynamics in a deterministic dynamical system, the 0–1 test for chaos retains the advantage, over the traditional methods for detecting chaos (using the maximal Lyapunov exponent), of displaying reduced sensitivity to measurement noise [61]. Briefly, for the first *n* = 1, …, *n*_max_ = 1000 samples of the input signal and *N*_*c*_ = 100 values of *c* chosen randomly in the interval (0, *π*), we evaluated the translation variables

$$p_{c}\left(n\right)=\overset{n}{\underset{j=1}{\sum }}V(j)\text{cos}\left(jc\right)\text{ and }q_{c}\left(n\right)=\sum_{j=1}^{n}V\left(j\right)\text{sin}\left(jc\right),$$ (2)

where *V* is the amplitude of the input signal. Secondly, considering the presence of measurement noise, we quantified for $n\leq \frac{n_{\text{max}}}{10}=n_{\text{cut}}$ the damped mean squared displacement of the translation variables, as follows

$$D_{c}^{*}\left(n\right)=M_{c}\left(n\right)-{\left(EV\right)}^{2}\frac{1-\text{cos}nc}{1-\text{cos}c}+h\;\cdot {\left(EV\right)}^{2}\text{sin}\left(\sqrt{2}n\right),$$ (3)

where *M*_*c*_*(n)* is the mean squared displacement of the translation variables, defined as

$$M_{c}\left(n\right)=\underset{N\to \infty }{\text{lim}}\frac{1}{N}\overset{N}{\underset{j=1}{\sum }}{\left[p_{c}\left(j+n\right)-p_{c}\left(j\right)\right]}^{2}+{\left[q_{c}\left(j+n\right)-q_{c}\left(j\right)\right]}^{2},$$

*EV* is the expectation of *V*, while parameter *h* was defined based upon optimization of the outcome of the test across a subset of 12 MER trajectories in PD and 12 MER trajectories in OCD. We next computed the strength of correlation of $D_{c}^{*}\left(n\right)$ with linear growth as

$$K_{c}=corr\left(\xi ,\Delta \right)=\frac{\text{cov}\left(\xi ,\Delta \right)}{\sqrt{\text{var}\left(\xi \right)\text{var}\left(\Delta \right)}}\in \left[-1,1\right],$$ (4)

where ***ξ*** = [1 2 ⋯ *n*_cut_] and $\Delta =\left[D_{c}^{*}\left(1\right)\;D_{c}^{*}\left(2\right)\;\cdots \;D_{c}^{*}\left(n_{\text{cut}}\right)\right]$. The outcome of the test, *K*_t_, was given by

$$K_{t}=median\;\left(K_{c}\right)$$ (5)

A test outcome, *K*_t_ < 0.1 indicated the presence of regular dynamics [62], i.e., the presence of significant cross-frequency coupling.

With respect to parameter determination, we used 1s (i.e., 1000 samples) of the input signal, since this value yielded the best trade-off between low computational cost and optimal outcome of the test. In addition, *N*_*c*_ = 100 different values of *c* have been proven to constitute an appropriate variable selection in [30].

The performance of the 0–1 test outcome was compared with the performance of an alternative measure of cross-frequency coupling, the *modulation index*. This index is based on the Kullback-Leibler (KL) distance between two distributions and its calculation involves application of the Hilbert transform combined with linear band-pass filtering [26, 63].

### Model-based control strategy for the identification of the optimal stimulation protocol

#### The phase-reduced model

We used a previously published stochastic phase-reduced model [40], inclusively allowing for the phase-response dynamics of a bursting neuron in both weak and strong perturbation regimes [46, 47]. The phase-reduced model is described by the following Ito stochastic differential equation (derivation of Eq (6) is provided in S1 Text):

$$\frac{\text{d}\phi }{\text{d}t}=\omega +Kr\text{sin}\left(2\pi \left(\psi -\phi +a\left(K,r\right)\right)\right)+v_{\text{C}}+\left(\sigma_{\text{I}}R_{\text{I}}\left(\phi \right)+\sqrt{D_{\text{C}}}\right)\;\xi \left(t\right)+\frac{\sigma_{\text{I}}}{2}{R^{\prime }}_{\text{I}}\left(\phi \right)\left(\sigma_{\text{I}}R_{\text{I}}\left(\phi \right)+\sqrt{D_{\text{C}}}\right)+\Delta \left(\phi ,\beta \right)\underset{k}{\sum }\delta \left(t-\tau_{k}\right)$$ (6)

where $v_{C}\approx \sigma_{\text{C}}{}^{2}\overset{\infty }{\underset{0}{\int }}ds\;C\left(s\right)\overset{1}{\underset{0}{\int }}d\phi \;{R^{\prime }}_{\text{C}}\left(\phi \right)\;R_{\text{C}}\left(\phi -\omega s\right)$ and $D_{\text{C}}\approx \sigma_{\text{C}}{}^{2}\overset{\infty }{\underset{-\infty }{\int }}ds\;C\left(s\right)\overset{1}{\underset{0}{\int }}d\phi \;R_{\text{C}}^{}\left(\phi \right)\;R_{\text{C}}\left(\phi -\omega s\right)$. Considering a rectangular stimulation waveform, parameter *β* may be expressed as: *β* = *wI*_0_ / *C* [64], where *w* represents the stimulus pulse width (expressed in *μs*), *I*_0_ is the stimulus current amplitude (expressed in *A*) and *C* = 1*μ*F/cm^2^.

Variable *ϕ* ∈ [0,1) denotes the oscillator’s phase, *ω* is its natural frequency, while *K*, *r* and *ψ* symbolize the coupling strength, the degree of synchrony and the mean phase of the oscillators, respectively, in the surrounding neural population. These parameters were evaluated based on the processing of the MERs, as described in [28, 40]. Parameter *α* represents the phase shift inherent to nonlinear coupling. This parameter was considered equal to ¼, so as to better capture the partially synchronous quasiperiodic dynamics (0 < *r* < 1) of the subthalamic neuronal activity in the pathological state [28, 65]. *ξ*(*t*) is the zero mean Gaussian white noise, added independently to the oscillator, and *η*(*t*) is the colored (common) noise with zero mean, unitary variance and autocorrelation function *C*(*t*). Parameters *σ*_I_ and *σ*_C_ denote the intensity of independent and common noise, and were determined based on the spiking activity and the LFP signal component, respectively [40]. Phase sensitivity functions *R*_I_(*ϕ*) and *R*_C_(*ϕ*) were evaluated according to [40, 46, 66]. Function *Δ*(*ϕ*, *β*) represents the phase response curve (PRC) to a single (DBS) impulse and was evaluated according to [47] (S1 Fig). Values of *β* were appropriately scaled according to the size of perturbations upon which the PRC was constructed. Variable *τ*_*k*_ denotes the input times (0 ≤ *k* < ∞). We considered that the inter-impulse interval (IPI) *Δτ*_*n*_ = *τ*_*n*+1_ − *τ*_*n*_ obeys a Poisson distribution with parameter *λ* and that stimulus trains have a mean frequency *f* (expressed in Hz).

We emphasize that the MERs used for the estimation of model parameters are exclusively those for which cross-frequency coupling and/or bursting activity was identified, as described in the previous section. Through this assignment, phase model (6) is being appropriately elaborated to capture the regular dynamics of pathological neuronal activity, in addition to simulating the effect of stimulation on this activity.

The recorded neuronal activity had to follow a bursting-like pattern in order to be compatible with the model, which is a bursting neuron model. Essentially, the first part of the closed-loop scheme is crucial for model parameter estimation in the second part.

After solving phase Eq (6), we employed the *Perron-Frobenius operator*, P, in order to extract the stochastic phase map from one stimulus cycle to the next:

$$p_{n+1}\left(\phi \right)=P\;p_{n}\left(\phi \right),$$ (7)

where *p*_*n*_(*ϕ*) and *p*_*n*+1_(*ϕ*) are the densities of the phases at the time of the *nth* and (*n* + 1)*th* impulses, respectively [40]. By discretizing the phase into *m* = 500 bins, operator P was approximated using a *m* × *m transition matrix* (or stochastic kernel), ***A*** = [*α*_*ij*_]. The iterative process (7) converges to the steady-state phase distribution, i.e., the invariant density, *p*_*st*_(*ϕ*). The invariant density vector, $p_{st}\in {\mathbb{R}}_{\geq 0}^{m}$, is the eigenvector corresponding to the dominant (unit) eigenvalue of the transition matrix. In accordance with [40], we assessed the variance of the elements of the invariant density vector, $\sigma_{p_{st}}^{2}$, as a quantity inversely related to the desynchronizing effect, but potentially also to clinical effectiveness of stimulation. We may express this variance in terms of the Euclidean norm of ***p***_***st***_, as

$$\sigma_{p_{st}}^{2}=\frac{1}{m}\|p_{st}-\mu_{p_{st}}1{\|}_{2}^{2},$$ (8)

where $\mu_{p_{st}}$ is the mean of the elements of the invariant density vector and ***1*** is the *m* by 1 vector of ones. It should be noted that we did *not* assess the variance of the phase variable, *ϕ*, which would be analogous to the desynchronizing effect of stimulation. Rather, we wanted to place emphasis on the properties of the derived largest eigenvector of the transition matrix by assessing the variance of its elements. Identification of the optimal stimulation protocol was based on minimization of the cost function

$$F\left(x\right)=\frac{1}{m}\|p_{st}-\mu_{p_{st}}1{\|}_{2}^{2}+gP,$$ (9)

where ***x*** is the vector of stimulation parameters, i.e., ***x*** = [*x*_1_ *x*_2_ *x*_3_ *x*_4_] = [*w I*_0_ *f λ*], and *P* = *I*_0_^2^*wf*·*R* is the stimulation power [67]. Parameter *g* is a penalizing scalar (we set *g* = 0.25), while *R* represents the electrode impendance (we considered R = 1000Ω).

#### Model-based derivative-free optimization

We considered the determination of the *d* = 4 optimal stimulation parameters for minimum energy desynchronizing control of neuronal activity as a constrained optimization problem defined as:

$${\text{min}}_{x\in {\mathbb{R}}_{}^{4}}F\left(x\right)$$ (10)

subject to 30 ≤ *x*_1_ ≤ 210,

$$0.001\;\leq x_{2}\leq \;0.004,$$

$$20\;\leq x_{3}\leq \;150,$$

$$3\;\leq x_{4}\leq \;30$$

Determination of the pulse-width constraints was based on evidence that pulse durations lower than 60 *μ*s may lead to increased selectivity of stimulation, i.e., activation of the targeted neural elements without activation of distant pyramidal tract fibers, and therefore possibly also to an increased therapeutic window of DBS [68].

Cost function (9) is expected to exhibit non-smooth behavior. We therefore employed a clever derivative-free optimization algorithm, in particular a model-based pattern search method guided by *simplex derivatives* (SID-PSM) [54, 55]. This algorithm belongs to the general class of direct search methods of directional type. Its distinguishing feature is the use of past objective function evaluations to improve the computational algorithmic efficiency.

Direct search methods of directional type are iterative algorithms, where the process of finding a new iterate (***x***_*k*+1_) can be organized in a search step and a poll step. The search step is optional, not required to ensure the convergence of the algorithm, and typically used to improve the numerical efficiency. In SID-PSM, past function evaluations are used to compute a local *quadratic model* of the objective function, which is minimized in a region of interest (a ball around the current iterate ***x***_*k*_). The corresponding minimizer is then evaluated for the original objective function and, if it decreases the value of the current iterate *F*(***x***_*k*_), it is accepted as the new iterate ***x***_*k*+1_. In this case, the iteration is declared as successful and the poll step is skipped.

If the search step fails in obtaining a better point, the algorithm will obligatorily perform the poll step, where a local search around the current best point ***x***_*k*_ is considered by evaluating the feasible points belonging to the poll set *P*_*k*_ = {*x*_*k*_ + *α*_*k*_*d*_*k*_: *d*_*k*_ ∈ *D*_*k*_}. In this case *α*_*k*_ represents a step size parameter and *D*_*k*_ a set of directions with good geometrical properties, typically corresponding to a positive generating set [69]. This evaluation process is opportunistic, meaning that a new point is accepted as a new iterate once it decreases the value of the objective function, without evaluating the remaining poll points. Thus, the order by which the directions are tested is relevant for the algorithmic performance. Using previous evaluated points, SID-PSM computes a *descent indicator*, based on simplex derivatives, and directions are tested according to the angle that they make with this descent indicator. If a better point is found during this testing procedure, the new point is accepted as a new iterate ***x***_*k*+1_, and the iteration is declared as successful. Otherwise, ***x***_*k*+1_ = ***x***_*k*_ and the iteration will be unsuccessful.

At the end of each iteration, the step size is updated: decreased at unsuccessful iterations and maintained or increased for successful ones. Points evaluated during both search and poll steps at iteration *k* are stored in a list, *X*_*k*_, allowing the computation of the quadratic models and of the descent indicators at no further expense in terms of function evaluations.

#### Incorporating quadratic models

Given a sample set ${Y^{\prime }}_{k}=\left\{{y^{\prime }}_{k}^{0},\;{y^{\prime }}_{k}^{1},\ldots ,\;{y^{\prime }}_{k}^{p_{k}}\right\}\subseteq X_{k}$ (where ${y^{\prime }}_{k}^{0}=x_{k}$), a quadratic polynomial basis *e* = {*e*_0_(*x*), *e*_1_(*x*), …, *e*_*q*_(*x*)} and a quadratic polynomial model $m\left({y^{\prime }}_{k}\right)=\alpha_{k}^{\text{T}}e\left({y^{\prime }}_{k}\right)$, the condition for quadratic polynomial interpolation can be expressed as

$$M\left(e,{Y^{\prime }}_{k}\right)\;\alpha_{k}=F({Y^{\prime }}_{k}),$$ (11)

where

$$M\left(e,{Y^{\prime }}_{k}\right)=\left[\begin{array}{cccc} e_{0}\left(x_{k}^{}\right) & e_{1}\left(x_{k}\right) & \cdots & e_{q}\left(x_{k}\right)\\ e_{0}\left({y^{\prime }}_{k}^{1}\right) & e_{1}\left({y^{\prime }}_{k}^{1}\right) & \cdots & e_{1}\left({y^{\prime }}_{k}^{1}\right)\\ \vdots & \vdots & \vdots & \vdots \\ e_{0}\left({y^{\prime }}_{k}^{p_{k}}\right) & e_{1}\left({y^{\prime }}_{k}^{p_{k}}\right) & \cdots & e_{q}\left({y^{\prime }}_{k}^{p_{k}}\right) \end{array}\right]\text{ and }F\left({Y^{\prime }}_{k}\right)=\left[\begin{array}{c} F\left(x_{k}\right)\\ F\left({y^{\prime }}_{k}^{1}\right)\\ \vdots \\ F\left({y^{\prime }}_{k}^{p_{k}}\right) \end{array}\right].$$

System (11) is determined, if *p*_*k*_ = *q* = (*d* + 1)(*d* + 2) / 2 − 1, overdetermined, if *p*_*k*_ > *q*, and underdetermined, if *p*_*k*_ < *q*. The quadratic polynomial model may also be written as

$$m\left({y^{\prime }}_{k}\right)=c_{k}+g_{k}^{\text{T}}{y^{\prime }}_{k}+\frac{1}{2}{y^{\prime }}_{k}^{\text{T}}H_{k}{y^{\prime }}_{k},$$ (12)

where ***g***_*k*_ and ***H***_*k*_ represent the gradient and the Hessian of the model, respectively. The quality of the quadratic model as approximation to the original function is strongly dependent on the norm of ***H***_*k*_. Thus, when a reasonable number of points is already available, but system (11) is still underdetermined (*d* + 1 ≤ *p*_*k*_ < *q*), a minimum Frobenius norm (MFN) solution is computed by minimizing the Frobenius norm of the Hessian ***H***_*k*_ subject to the interpolation conditions:

$$\text{min}\frac{1}{4}\|H_{k}{\|}_{F}^{2}$$ (13)

subject to $c_{k}+g_{k}^{\text{T}}\left({y^{\prime }}_{k}^{i}\right)+\frac{1}{2}{\left({y^{\prime }}_{k}^{i}\right)}^{\text{T}}H_{k}\;\left({y^{\prime }}_{k}^{i}\right)=F\left({y^{\prime }}_{k}^{i}\right),\;i=0,1,\ldots ,p_{k}.$

If *q* < *p*_*k*_ ≤ (*d* + 1)(*d* + 2) − 1, a regression quadratic model is considered by solving the problem:

$$\text{min}\frac{1}{2}\|M\left(e,{Y^{\prime }}_{k}\right)\alpha_{k}-F\left({y^{\prime }}_{k}\right){\|}_{}^{2}.$$ (14)

When there are more points available than the ones required to build the overdetermined model, a subset of *X*_*k*_ is selected, with 80% of the points chosen near to the current iterate, ***x***_*k*_, and the remaining 20% chosen far from it. The search step will be defined by minimizing the quadratic model in

$$L\left(x_{k};\Delta_{k}\right)=\left\{x\in R^{d}:\left\|x-x_{k}\right\|\leq \Delta_{k}\right\},$$

a ball centered at ***x***_*k*_, with radius $\Delta_{k}=\sigma_{k}\alpha_{k-1}\underset{d\in D_{k-1}}{\text{ max}}\left\|d\right\|$. Parameter *σ*_*k*_ equals to 1, if the previous iteration was unsuccessful, or 2, otherwise.

#### Incorporating descent indicators

At the beginning of each poll step, a sample set $Y_{k}=\left\{y_{k}^{0},\;y_{k}^{1},\ldots ,\;y_{k}^{r_{k}}\right\}\subseteq X_{k}$ (where $y_{k}^{0}=x_{k}$) with some desirable geometric properties may be identified. This sample set should be part of a ball of the same or larger radius of the smallest ball enclosing the poll set *P*_*k*_. The simplex gradient of *F* at ***x***_*k*_, $g_{k}=\nabla_{S}{}_{{}_{k}}F\left(x_{k}\right)$, is the solution of the following system

$$S_{k}^{\text{T}}g_{k}=\delta_{k}\left(F;S_{k}\right),$$ (15)

where $S_{k}=\left[y_{{}_{k}}^{1}-x_{k}\cdots y_{k}^{r_{k}^{}}-x_{k}\right]$ and $\delta_{k}\left(F;S_{k}\right)={\left[F\left(y_{k}^{1}\right)-F\left(x_{k}\right)\cdots F\left(y_{k}^{r_{k}}\right)-F\left(x_{k}\right)\right]}^{\text{T}}$. Again, this system allows determined (*r*_*k*_ = *d*), underdetermined (*r*_*k*_ < *d*) or overdetermined (*r*_*k*_ > *d*) solutions. Underdetermined and overdetermined forms of simplex gradients are computed by

$$\nabla_{S_{k}}F\left(x_{k}\right)=V_{k}\Sigma_{k}{}^{-1}U_{k}{}^{\text{T}}\delta_{k}\left(F;S_{k}\right)/\Delta_{k},$$ (16)

where $U_{k}\Sigma_{k}V_{k}{}^{\text{T}}$ is the singular value decomposition of the scaled matrix $S_{k}^{\text{T}}/\Delta_{k}$ and Δ_*k*_ is defined as in the previous section.

Importantly, the quality of simplex gradients as approximations to some form of real function derivatives has been established even in the non-smooth case [70] and depends on the geometrical properties of the sample set. SID-PSM uses the geometrical notion of Λ-*poisedness* to determine the quality of the geometry of the sample set and considers that a sample set *Y*_*k*_ is Λ-poised, if $\left\|\Sigma_{k}^{-1}\right\|\leq \Lambda$, for some positive constant Λ. Thus, the negative simplex gradient $-\nabla_{S}{}_{{}_{k}}F\left(x_{k}\right)$ may be considered as a direction of potential descent, namely this gradient constitutes a descent indicator, which is used for ordering the poll vectors. In particular, the polling procedure will start by first testing the poll vectors that make the smallest angle with the negative simplex gradient. S2 Fig displays an exemplary record of the objective function value in terms of the total number of function evaluations for SID-PSM.

### Assessment of a possible correlation of the invariant density measure with clinical effectiveness of stimulation in OCD

In order to assess a possible correlation of the invariant density measure with clinical effectiveness of stimulation in treatment-refractory OCD, we evaluated $\sigma_{p_{st}}^{2}$ simulating the application of regular 130 Hz stimulation and based upon the model parameters estimated for two subsets of recordings: a total of 39 MERs of subthalamic neuronal activity acquired during DBS for OCD and characterized by a high mean discharge rate (39.7 ± 14.71 Hz), a high intraburst frequency and a short interburst interval (*μ*_ISI_ = 0.0289 ± 0.0114s, *Var*_ISI_ = 0.0038 ± 0.0056) vs. a total of 39 MERs of subthalamic neuronal activity characterized by a low mean discharge rate (13.53 ± 7.13Hz), a low intraburst frequency and a long interburst interval (*μ*_ISI_ = 0.1072 ± 0.093s, *Var*_ISI_ = 0.0265 ± 0.0542). This specific approach was based on indications correlating the efficacy of standard STN-DBS for OCD with locations of neuronal activity characterized by a high discharge rate and intraburst frequency, and a short interburst interval [50]. Statistical significance was determined by means of the Mann—Whitney *U* test.

## Results

### Noise-resistant assessment of nonlinear coupling

**Fig 2:**
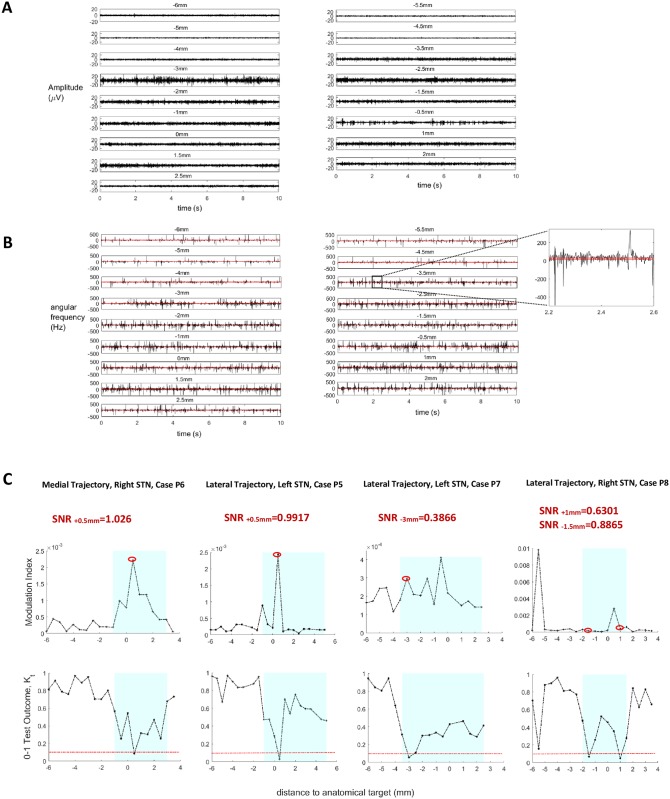
Performance comparison between the modulation index and the 0–1 test outcome. (A) Exemplary LFP signals acquired along the lateral MER trajectory for the identification of the right STN in a case of PD (P8). (B) The respective instantaneous angular frequency series are characterized by a high rate of singularities. Here, angular frequency is defined as the derivative of the (unwrapped) phase estimated by means of the Hilbert transform after linear band-pass filtering the signal between 13 and 30 Hz. By setting as thresholds the limits of the band-pass filter (red horizontal lines), slip occurences are identified at the time points of threshold crossing (inset). (C) Comparative assessment of the modulation index and the 0–1 test outcome in four representative cases of PD. Results obtained by means of both measures are in good agreement in case of a relatively high signal to noise ratio (SNR) (cases P5 and P6: identification of significant cross-frequency coupling at +0.5 mm). On the contrary, in the presence of increased noise levels, the high rate of artificial phase slips renders the calculation of cross-frequency coupling by means of the modulation index unreliable. In particular, the index fails to discriminate sites with significant non-linear coupling from sites without, in case of a low SNR (cases P7 and P8: significant cross-frequency coupling at -3 mm and at -1.5 mm/+1 mm, respectively, identified only by means of the 0–1 test for chaos).

Fig 2A illustrates exemplary LFPs acquired intraoperatively along a particular MER trajectory. For assessment of the modulation index, after linear band-pass filtering the LFP signals between 13 and 30 Hz, the respective phase series is estimated by means of the Hilbert transform. As illustrated in Fig 2B, the derivative of the phase so estimated, i.e. the instantaneous angular frequency, is characterized by a high rate of singularities reflecting a high rate of artificial phase slips. At a singularity (or phase slip), the instantaneous angular frequency reaches values that exceed the limits imposed by the band-pass filter [71] (inset; Fig 2B). The high rate of artificial phase slips is probable to render the calculation of cross-frequency coupling unreliable, particularly in the presence of increased noise levels. On the contrary, a phase-reconstruction-free method does inherently not suffer from the ambiguity associated with phase singularities. This fact is demonstrated through four representative cases in Fig 2C. While results obtained by means of the modulation index and the test outcome are in good agreement in case of a relatively high signal to noise ratio (SNR) (cases P5, P6; Fig 2C), the modulation index fails to discriminate sites with significant non-linear coupling from sites without, in case of a low SNR (cases P7, P8; Fig 2C).

**Fig 3:**
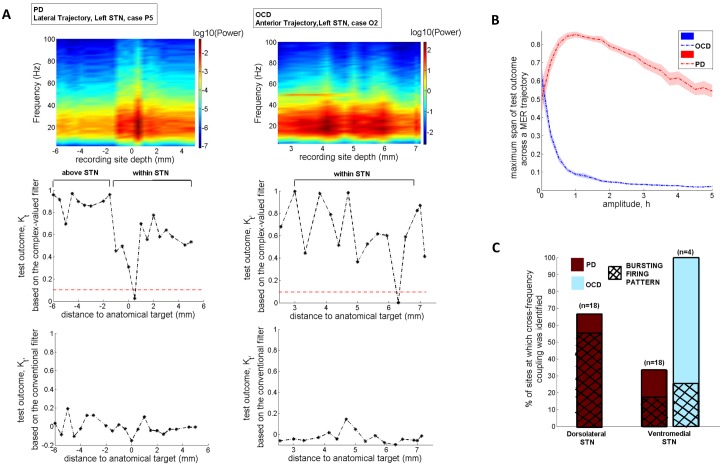
Exemplary and cumulative results of the methodology applied for the assessment of cross-frequency coupling as a biomarker for feedback control. (A) Exemplary results corresponding to a case of PD and a case of OCD. Figures at the top display the power spectrum of the filtered signals (13-30Hz) along each exemplary trajectory. (B) Determination of parameter *h* (Eq (3)), based upon optimization of the outcome of the 0–1 test for chaos across a subset of 12 MER trajectories in PD and 12 MER trajectories in OCD. According to the results, in case of PD, sensitivity to measurement noise had to be further decreased by assigning a unitary value to parameter *h*. (C) Cross-frequency coupling was identified at at least 1 site within the STN of each patient with PD (total:18 MERs). Approximately 67% of these sites was located at the dorsal border of the STN, while at 72.2% of these sites neuronal activity followed a bursting or burst-like firing pattern and was considered for further processing in the phase-reduced bursting neuron model. Contrary to the case of PD, cross-frequency coupling was identified within the STN of only 2 patients with treatment-refractory OCD (total:4 MERs).

In the two representative cases of Fig 3A, employment of the 0–1 test for chaos following the application of the complex valued-filter, singled out sites with significant nonlinear coupling between beta and high-frequency activity (indicated by a test outcome smaller than 0.1). Conversely, following the application of a conventional Butterworth band-pass filter, the 0–1 test for chaos did not discriminate sites with significant non-linear coupling from sites without. This result was corroborated across the total of the MER trajectories examined and highlighted the robustness of the two-part technique, i.e., the *combined* application of the complex-valued filter and the 0–1 test for chaos. In case of PD, reduced sensitivity to measurement noise in the 0–1 test for chaos was warranted by assigning a positive value to parameter *h* in Eq (3) (*h* = 1, Fig 3B). On the contrary, this assignment did not prove to be a prerequisite in case of OCD (*h* = 0, Fig 3B).

#### Nonlinear coupling may be a reliable biomarker for feedback control in case of STN-DBS for PD

Cross-frequency coupling was identified at a total of 18 MERs—sites within the STN of 8 patients with PD (case P1: 2 sites; case P2: 1 site; case P3: 3 sites; case P4: 2 sites; case P5: 3 sites; case P6: 2 sites; case P7: 1 site; case P8: 4 sites). Approximately 67% of these sites (n = 12) was located at the dorsal border of the STN (Fig 3C). These results are rather predictable given that beta-HFO coupling is closely correlated with the pathophysiology of PD and strongest at the dorsal border of the STN [20–24]. They further corroborate the potential appropriateness of nonlinear coupling between beta and high-frequency neuronal activity as a biomarker for feedback control in PD. Neuronal activity at 13 out of the 18 sites with cross-frequency coupling followed a bursting or burst-like firing pattern (case P1: 1 site; case P2: 1 site; case P3: 3 sites; case P4: 1 site; case P5: 3 sites; case P6: 2 sites; case P7: 1 site; case P8: 1 site). These sites were considered for further processing in the bursting neuron model. At the remaining 5 sites a rather irregular firing pattern was observed, and therefore these sites were excluded from subsequent analysis.

#### Nonlinear coupling may display subject-specific applicability as a biomarker for feedback control in case of STN-DBS for OCD

Contrary to the case of PD, cross-frequency coupling was identified at only 4 MERs—sites within the STN of 2 patients with OCD (case O2: 2 sites; case O3: 2 sites) (Fig 3C). The latter fact may be attributable to the lower number of acceptable MER trajectories in case of STN—DBS for OCD. Otherwise, it implies that nonlinear coupling between beta and high-frequency activity may not consistently be an appropriate biomarker for feedback control in closed-loop STN-DBS for treatment-refractory OCD [25] and that an alternative biomarker should, therefore, additionally be considered (Fig 1B). For this reason, on the basis of evidence pointing to a correlation of subthalamic bursting neuronal activity, characterized by certain features, with symptom severity and stimulation efficacy in OCD [50], we assessed, for the remaining of the cases wherein no cross-frequency coupling was identified, the presence of bursting neuronal activity with specific characteristics, i.e., a short interburst interval and a high intraburst frequency (*μ*_ISI_ = 0.0242 ± 0.0113s, *Var*_ISI_ = 0.0059 ± 0.0083). Specifically, we considered for further processing a total of 12 MERs (case O1: 1 site; case O2: 2 sites; case O3: 2 sites; case O4: 1 site; case O5: 1 site; case O6: 1 site; case O7: 2 sites; case O8: 2 sites).

### Performance of the model-based control strategy in terms of efficiency, selectivity of stimulation and computational cost

**Fig 4:**
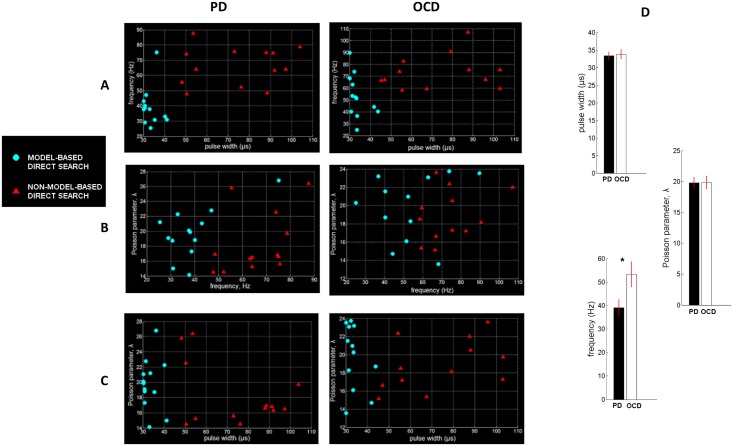
Implementation results of the model-based vs. the non-model-based approach. The model-based direct search method (SID-PSM) was compared with a non-model-based direct search method ([72]; Matlab, Mathworks, Natick, MA) in terms of the acquired parameters of stimulation, based on 13 MERs acquired during STN-DBS surgery for advanced PD and 12 MERs acquired during STN-DBS surgery for treatment-refractory OCD. For each site, we acquired five sets of parameter values, by means of each distinct solver, and assessed the respective mean values displayed in (A)-(C). The current amplitude, after application of both optimization procedures, was consistently maintained at its minimal value (*I*_0_ = 0.001*A*). (D) Mean values of the optimal stimulation settings following the application of the model-based direct search method. The mean optimal stimulation frequency proved to be significantly higher in case of OCD compared with PD (*p = 0.02, Mann—Whitney *U* test). Errorbars indicate standard error mean.

The performance of the model-based direct search method (SID-PSM) in the determination of the optimal parameters of stimulation in cases of PD and OCD was compared with the performance of a non-model-based generalized pattern search method ([72]; Matlab, Mathworks, Natick, MA), in terms of the resulting values of the invariant density, stimulation power and total computation time. At each site, we acquired five evaluations of optimal stimulation parameters by means of each distinct solver and assessed the respective mean values illustrated in Fig 4A–4C. In both optimization procedures, current amplitude was consistently maintained at its minimal value (*I*_0_ = 0.001*A*). The optimal pulse width determined by means of the model-based direct search method in case of PD proved to be equal to 33.36 ± 1.06 *μ*s (mean ± standard error mean) and in case of OCD, equal to 33.75 ± 1.29 *μ*s (mean ± standard error mean) (Fig 4D). Given that pulse durations lower than 60 *μ*s have been associated with increased selectivity of stimulation [68], this result indicates a potentially outstanding performance of the model-derived stimulation parameters in terms of selectivity of stimulation. We should further comment on the fact that, following the application of the model-based direct search method, the mean optimal stimulation frequency proved to be significantly higher in case of OCD compared with PD (*p*<0.05, Mann—Whitney *U* test), while a similar outcome was obtained with respect to the mean optimal pulse width and the mean optimal Poisson parameter (Fig 4D). We suggest that differences in the underlying pathophysiology [49, 50] may have led to the observed differences in optimal stimulation frequency in case of PD vs. treatment-refractory OCD. The derived mean optimal stimulation frequency of 39 ± 3.43 Hz in case of PD is in partial accordance with Class III evidence that STN-DBS at patient-specific frequencies in the range 31–100 Hz may improve motor Unified Parkinson’s Disease Rating Scale (mUPDRS) scores equally effectively with high-frequency stimulation in patients with PD [73].

**Fig 5:**
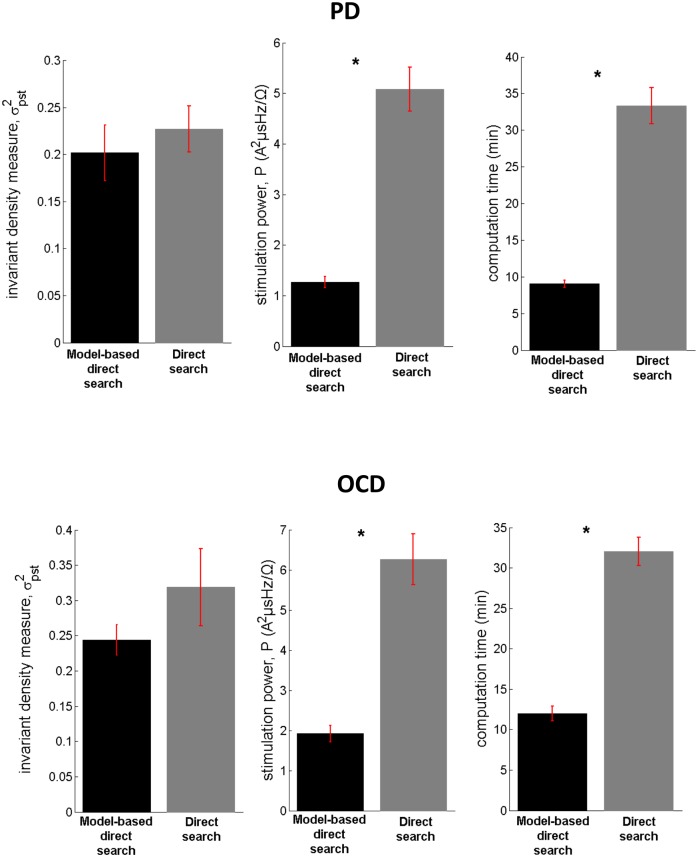
Performance of the model-based vs. the non-model-based approach in terms of efficiency of stimulation and computational speed. Comparison of the model-based direct search method (SID-PSM) with a non-model-based direct search method ([72]; Matlab, Mathworks, Natick, MA) corroborated a significantly higher performance of the former method, in terms of both stimulation power and computation time (*p<0.0001, Mann—Whitney *U* test), while an almost equivalent effect was observed on the invariant density measure (*p*_PD_ = 0.3299, Mann—Whitney *U* test). Errorbars indicate standard error mean.

Statistical analysis corroborated a significantly higher performance of the model-based direct search method, in terms of both stimulation power and computation time corresponding to the optimal stimulation settings, compared with the non-model-based generalized pattern search method (*p*<0.0001, Mann—Whitney *U* test), while an almost equivalent effect was observed on the invariant density measure (*p*_PD_ = 0.3299, *p*_OCD_ = 0.4705, Mann—Whitney *U* test) (Fig 5).

**Fig 6:**
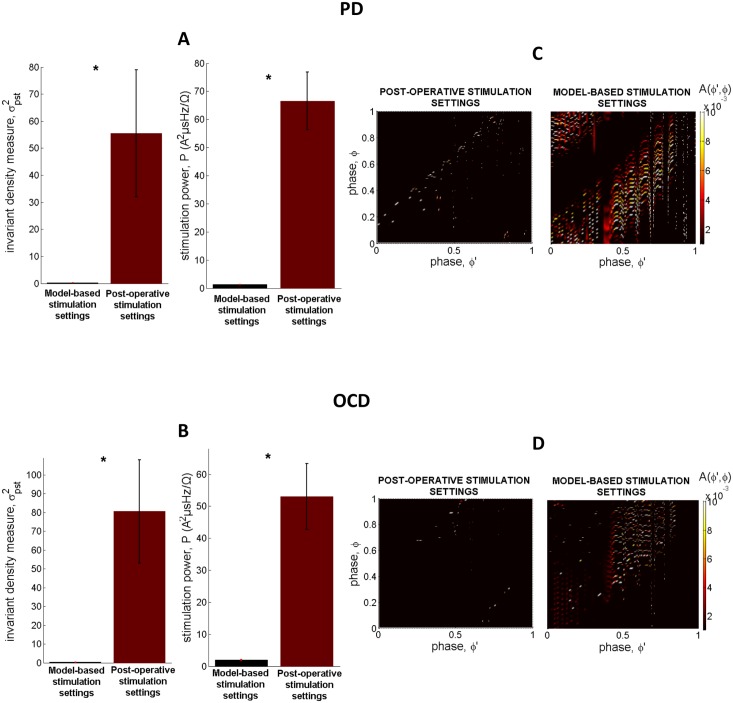
Performance of the model-based control strategy in terms of efficiency of stimulation. (A)-(B) Statistical analysis corroborated the ability of the model-based control strategy (combined application of the stochastic phase-reduced model and the model-based direct search method) to identify stimulation settings that yield significantly lower values of the invariant density measure and stimulation power compared with the respective values obtained by simulating application (through employment of the stochastic phase-reduced model) of the stimulation settings determined post-operatively, during the last follow-up of patients with PD and OCD (*p<0.0001, Mann—Whitney *U* test). Errorbars indicate standard error mean. (C)-(D) The depicted stochastic kernels were acquired by fitting the phase-reduced bursting neuron model to an exemplary set of MERs acquired during STN-DBS surgery for PD and OCD, respectively, and simulating application of the post-operative (left panels) vs. the model-based stimulation settings (right panels). The stronger desynchronizing effect on neuronal activity exerted by the model-based stimulation settings is qualitatively reflected in the intense form of the respective stochastic kernels.

The results corresponding to the stimulation settings determined by means of the model-based control strategy (combined application of the stochastic phase-reduced model and the model-based direct search method) were further compared with the results obtained by simulating application (through employment of the stochastic phase-reduced model) of the stimulation settings determined post-operatively, during the last follow-up of patients having undergone STN-DBS for PD or OCD (Tables 1 and 2). The comparison was performed in terms of the values of the invariant density measure and stimulation power. Statistical analysis corroborated the ability of the model-based control strategy to identify stimulation settings that yield significantly lower values of the invariant density measure and stimulation power compared with the respective values acquired by simulating application of the stimulation settings determined post-operatively (*p*<0.0001, Mann—Whitney *U* test) (Fig 6A and 6B). This result combined with the reported possible correlation of the invariant density measure with clinical effectiveness of stimulation in PD [40], but probably also in OCD (see next section), points to a potentially superior performance of the model-based stimulation parameters in terms of therapeutic and energy efficiency of stimulation. The differential desynchronizing effect on neuronal activity exerted by the model-based stimulation settings vs. the stimulation settings determined post-operatively is qualitatively reflected in the distinct form of the respective stochastic kernels (Fig 6C and 6D).

**Table 1:** Stimulation settings determined post-operatively during the last follow-up visit for patients with advanced PD.

case	Brain Hemi-sphere	pulse width (*μs*)	voltage (*V*)	frequency (*Hz*)
**P1**	Right	60	3.8	140
	Left	60	2.6	140
**P2**	Right	60	1.8	130
	Left	60	1.7	130
**P3**	Right	60	2.2	130
	Left	60	2	130
**P4**	Right	60	1.5	130
	Left	60	1.3	130
**P5**	Right	60	3.4	130
	Left	60	2.2	130
**P6**	Right	90	2.7	150
	Left	90	2.9	150
**P7**	Right	60	3.7	150
	Left	60	2.9	150
**P8**	Right	90	3.2	140
	Left	60	2.7	140

**Table 2:** Stimulation settings determined post-operatively during the last follow-up visit for patients with treatment-refractory OCD.

case	Brain Hemi-sphere	pulse width (*μs*)	voltage (*V*)	Frequency (*Hz*)
**O1**	Right	60	2	130
	Left	60	2	130
**O2**	Right	60	4	130
	Left	60	4	130
**O3**	Right	60	1.9	130
	Left	60	2	130
**O4**	Right	60	2.4	130
	Left	60	2.4	130
**O5**	Right	60	2	130
	Left	60	2	130
**O6**	Right	60	1.5	130
	Left	60	1.5	130
**O7**	Right	60	3	130
	Left	60	2.3	130
**O8**	Right	60	2	130
	Left	60	2	130

### Possible correlation between the invariant density measure and clinical effectiveness of stimulation in OCD

**Fig 7:**
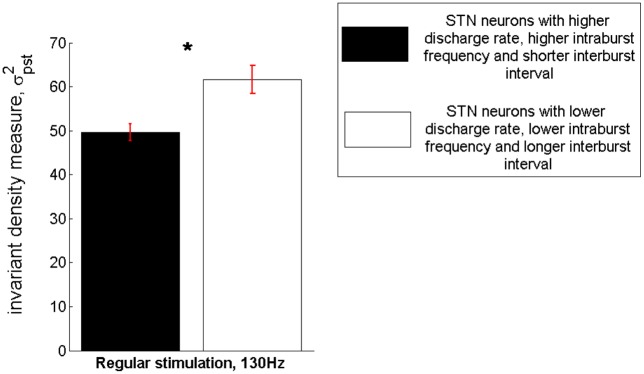
Possible correlation between the invariant density measure and clinical effectiveness of stimulation in OCD. Assessment of the mean ± standard error mean value of the invariant density measure based on a total of 39 MERs of subthalamic neuronal activity acquired during DBS surgery for treatment-refractory OCD and characterized by a high mean discharge rate (39.7 ± 14.71 Hz), a high intraburst frequency and a short interburst interval (μ_ISI_ = 0.0289 ± 0.0114s, Var_ISI_ = 0.0038 ± 0.0056) vs. a total of 39 MERs of subthalamic neuronal activity characterized by a low mean discharge rate (13.53 ± 7.13 Hz), a low intraburst frequency and a long interburst interval (μ_ISI_ = 0.1072 ± 0.093s, Var_ISI_ = 0.0265 ± 0.0542). The mean desynchronizing effect of standard 130Hz stimulation proved to be significanlty stronger in the former case compared with the latter (*p< 0.01, Mann—Whitney U test).

Fig 7 displays the results obtained by assessing the invariant density measure based on a total of 39 MERs of subthalamic neuronal activity acquired during DBS for OCD and characterized by a high discharge rate, a high intraburst frequency and a short interburst interval vs. a total of 39 MERs of subthalamic neuronal activity characterized by a low discharge rate, a low intraburst frequency and a long interburst interval. Remarkably, the desynchronizing effect of standard 130Hz stimulation proved to be significantly stronger in the former case compared with the latter (*p*<0.01, Mann—Whitney *U* test). This result points to a possible correlation of the invariant density measure with clinical effectiveness of stimulation in OCD, since values of this measure are proven to be lower at locations of neuronal activity that have been correlated with the best clinical outcome of STN-DBS for OCD [50].

## Discussion

Bikson et al. (2015) [74] remark: “Approaches using closed-loop stimulation are inherently *state* dependent and require *computational neurostimulation*.” Elaborating on this concept and considering the implications of the current approach, we make the following two key observations: first, though evidence about the pathophysiology of medically refractory movement and neuropsychiatric disorders remains to date to a large extent inconclusive, a growing body of basic and clinical work supports the important role of nonlinear coupling between beta and high-frequency activity in the pathophysiology of PD [75], thereby pointing to a possible utility of this measure as a state biomarker in closed-loop neuromodulation approaches for PD. Nevertheless, any attempt to reliably assess this biomarker should be made by carefully considering the presence of strong internal and measurement noise in the recorded neural activity. In this study, we presented an innovative technique drawn from dynamical systems theory guaranteeing low sensitivity to noise, and corroborated the presence of cross-frequency coupling in each case with advanced PD. Thereby, we provided indications for a possible appropriateness of this approach in optimization of closed loop neuromodulation systems.

Second, throughout this paper, we attempted to provide compelling evidence for the critical role of computational neurostimulation in closed-loop identification of novel stimulation protocols [56, 76]. The computational model employed operates on the principles of phase reduction and phase-resetting that are inherently characterized by simplicity and analytical tractability [48, 77–79], and further incorporates the dynamics of neuronal bursting activity that constitutes a hallmark of PD and OCD pathophysiology. In addition to the employment of the phase-reduced bursting neuron model, employment of direct search optimization based on quadratic modeling has significantly contributed to the performance of the presented approach. Crucially, since the quality of simplex gradients as approximations to the real derivatives of the objective function has been demonstrated in the non-smooth case [70], SID-PSM comprised an ideal choice for the problem under consideration, i.e., the non-smooth dynamics of the neuronal response to stimulation.

In previous work, we provided important indications for the realistic substructure of the stochastic phase-reduced model and further highlighted a possible correlation of the invariant density measure with clinical effectiveness of stimulation in PD [40]. By extending the latter result to the case of treatment-refractory OCD, we here prove that the proposed model-based control strategy holds the potential to exhibit remarkable performance in terms of therapeutic and energy efficiency of stimulation for both pathologic conditions. By yielding a mean optimal pulse width equal to ~33*μ*s, the model-based control strategy may further achieve outstanding performance in terms of selectivity of stimulation. Importantly, application of model-based direct search has been associated with a significantly higher computational speed compared with non-model-based derivative-free optimization.

There are several methodological considerations inherent to this work. Even though this study may provide a rigorous theoretical foundation for the design of a therapeutically- and energy-efficient closed-loop neuromodulation system, it should be pointed out that the stochastic model employed here does not capture large-scale neuronal interactions within key circuits involved in the pathophysiology of PD or OCD. Nonetheless, similar approaches have led to valid interpretations of the neuronal response to stimulation [80]. Another consideration is related to the potential effect of dopaminergic treatment on the performance of the proposed methodology. This assessment was not feasible in the current study, since the available data were acquired after a long period of withdrawal of medication. Third, clinical validation of the presented predictions should comprise a basic priority in future studies. Clinical validation may be possible once novel neural probes or systems offering the capability of concomitant DBS and microelectrode recording are introduced in clinical practice [81]. We also emphasize the necessity to test whether the findings of this study are replicated in macroelectrode recordings, and whether the model-based predictions are validated clinically based on current neuromodulation technologies [82]. Furthermore, modeling approaches similar to those proposed in this study may display greater fidelity in the framework of constant current stimulation. The transition from the use of constant-voltage to constant-current DBS devices is being motivated by the rationale that constant-current stimulation will accommodate for inter-patient and temporal fluctuations in the impedance of the tissue and electrode-tissue interface [83, 84].

Last, special mention should be made of further important factors for efficient closed-loop neuromodulation. These include the optimization of the stimulation waveform [85], of the circuit topology [86, 87] and of contact selection [88], as well as the incorporation of neurochemical control [89, 90] and the adjustment of closed-loop DBS systems to the phenotypical heterogeneity of movement and neuropsychiatric disorders [91, 92]. Cumulative research towards these directions may ultimately favor the optimization of less invasive, groundbreaking treatment options including closed-loop optogenetic control [93, 94].

## Supporting information

S1 Fig The phase response curve employed in the stochastic model (evaluated according to Mauroy et al. (2014) [47]). (TIF)

S2 Fig Progression of the model-based direct search method for a single trial (Central -4.3mm, Right STN, case O3). (A) Cost function minimization was achieved after a total of 13 iterations and approximately 38 function evaluations. According to the algorithm, optimal stimulation settings for this particular example included a pulse width of 30μs (B), a current amplitude equal to 1mA (C), a stimulation frequency of 60Hz (D) and a Poisson parameter equal to 13 (E).
(TIF)

S1 Text Supplementary information for “Algorithmic design of a noise-resistant and efficient closed-loop deep brain stimulation system: A computational approach”. (PDF)

S1 File Minimal underlying data set. (XLSX)
