# Supplementary figures and images for: Algorithmic design of a noise-resistant and efficient closed-loop deep brain stimulation system: A computational approach
Source: PLoS One. 2017 Feb 21;12(2):e0171458. doi: 10.1371/journal.pone.0171458 (PMC5319757; doi:10.1371/journal.pone.0171458)

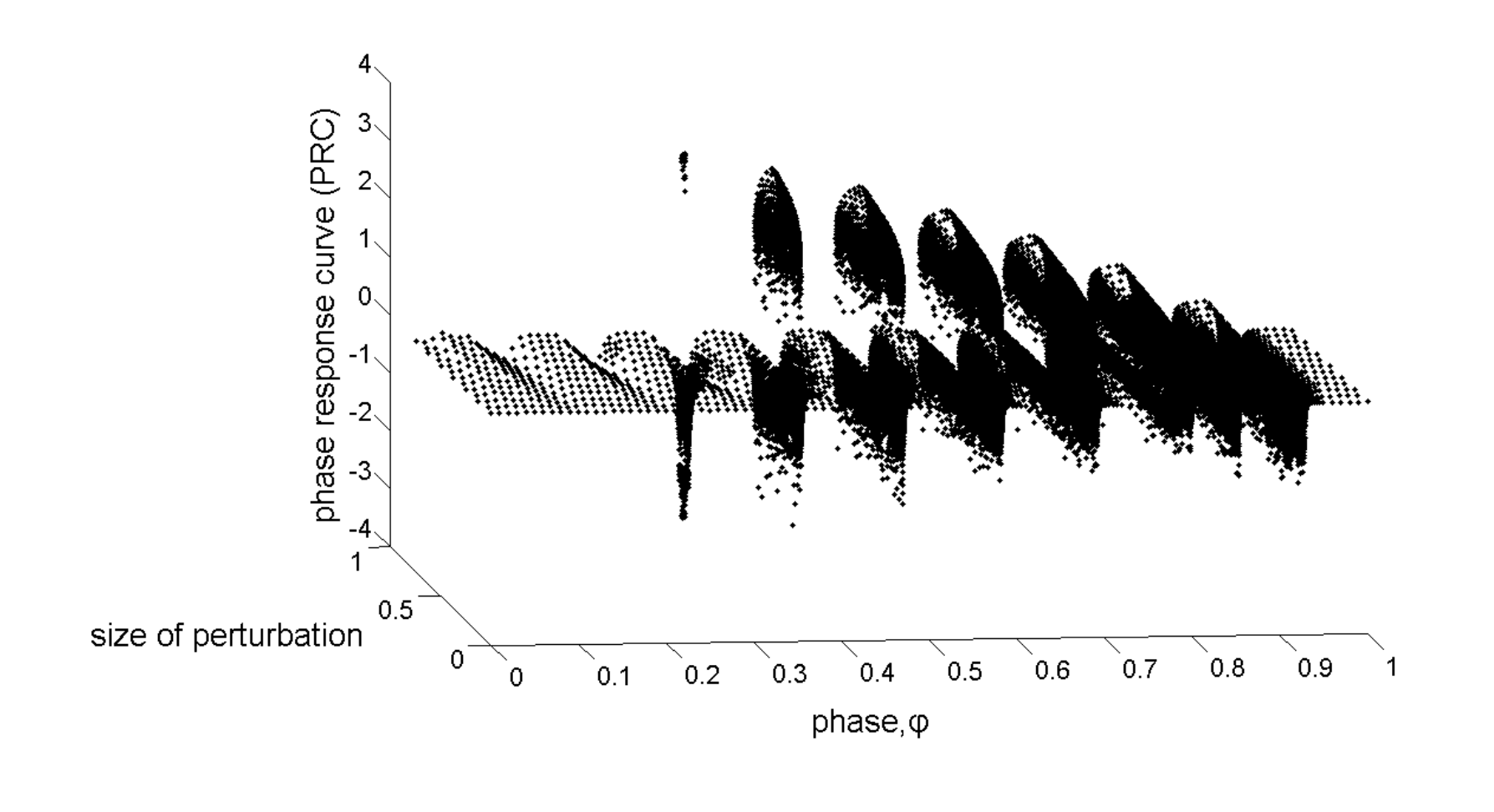

Supplement: S1 Fig — (TIF) [file pone.0171458.s001.tif]

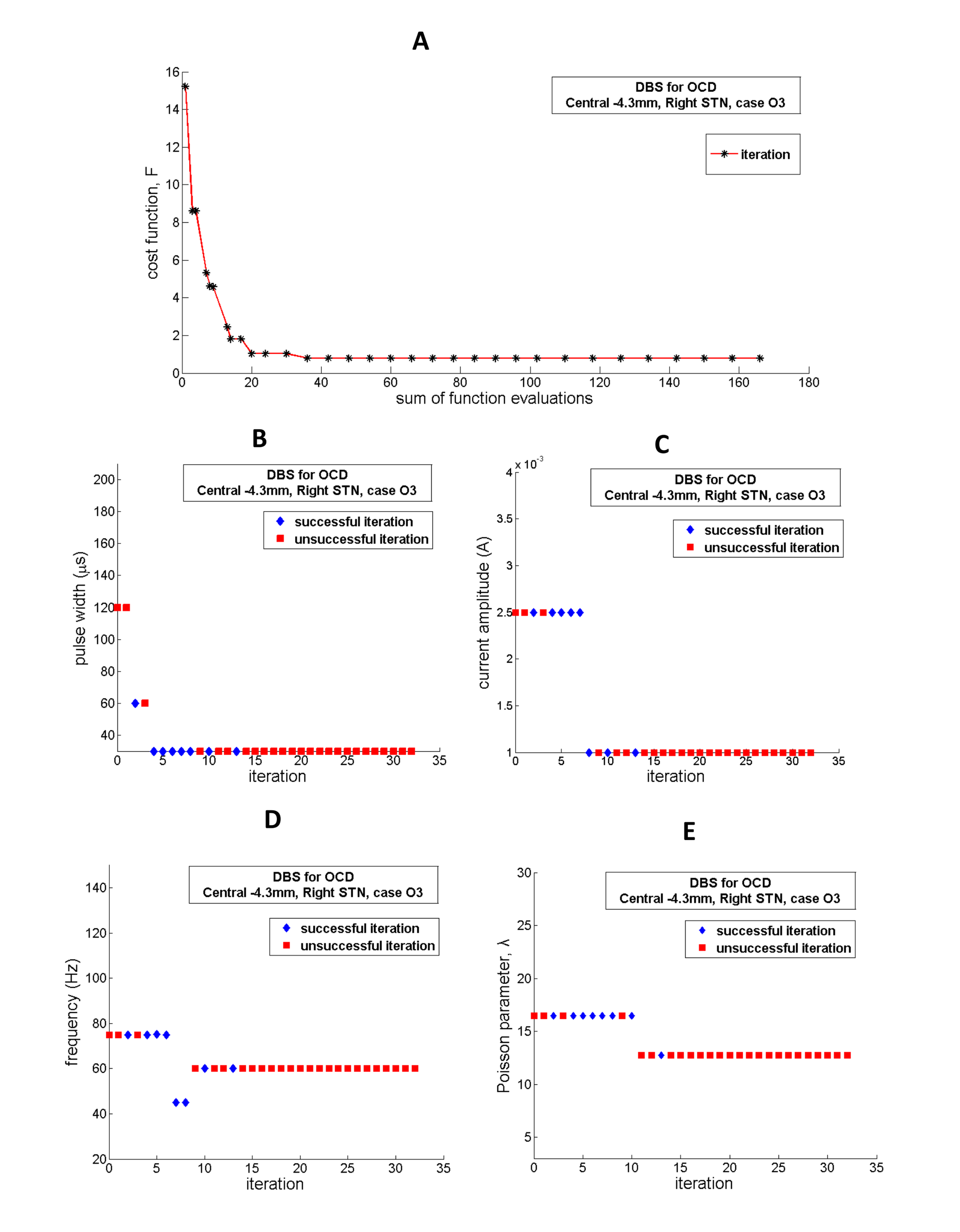

Supplement: S2 Fig — (A) Cost function minimization was achieved after a total of 13 iterations and approximately 38 function evaluations. According to the algorithm, optimal stimulation settings for this particular example included a pulse width of 30μs (B), a current amplitude equal to 1mA (C), a stimulation frequency of 60Hz (D) and a Poisson parameter equal to 13 (E). (TIF) [file pone.0171458.s002.tif]
