# Supplementary material for: Algorithmic design of a noise-resistant and efficient closed-loop deep brain stimulation system: A computational approach
Source: PLoS One. 2017 Feb 21;12(2):e0171458. doi: 10.1371/journal.pone.0171458 (PMC5319757; doi:10.1371/journal.pone.0171458)
Supplement: S1 Text — (PDF) [file pone.0171458.s003.pdf]

Supplementary Information for "Algorithmic Design of a Noise-Resistant and Efficient Closed-Loop Deep Brain Stimulation System: a Computational Approach"

Sofia D. Karamintziou[1,2]*, Ana Luísa Custódio[3], Brigitte Piallat[4,5], Mircea Polosan[5,6], Stéphan Chabardès[4,5,7], Pantelis G. Stathis[8], George A. Tagaris[9], Damianos E. Sakas[10], Georgia E. Polychronaki[1], George L. Tsirogiannis[1], Olivier David[4,5] and Konstantina S. Nikita[1]*

[1]School of Electrical and Computer Engineering, National Technical University of Athens, Athens, Greece

[2]Department of Mechanical Engineering, University of California, Riverside, CA, 92521 USA

[3]Department of Mathematics, FCT-UNL-CMA, Caparica, Portugal

[4]Univ. Grenoble Alpes, Grenoble Institut des Neurosciences, GIN, F-38000 Grenoble, France

[5]Inserm, U1216, F-38000 Grenoble, France

[6]Department of Psychiatry, University Hospital of Grenoble, Grenoble, France

[7]Department of Neurosurgery, University Hospital of Grenoble, Grenoble, France

[8]Department of Neurology, Mediterraneo Hospital, Athens, Greece

[9]Department of Neurology, 'G. Gennimatas' General Hospital of Athens, Athens, Greece

[10]Department of Neurosurgery, University of Athens Medical School, 'Evangelismos' General Hospital, Athens, Greece

*Corresponding authors

E-mails: skaram@biosim.ntua.gr (SDK) and knikita@ece.ntua.gr (KSN)

Derivation of the Stochastic Phase Model

We start from a well-established model of neuronal bursting, the Hindmarsh-Rose model for bursting **[1]**, allowing for the effect of neuronal coupling and noise perturbations, as well as for the effect of external stimulation. The corresponding oscillatory dynamics is defined by the following equations:

$$C\dot{V}_i = I^g(V_i,q) + I^b + a_\ell \sum_{j=1}^{N}(V_j - V_i) + w(V_i,t) + z(V_i,t) + I(V_i,t), \quad i = 1,...N \tag{1}$$

$$\dot{q} = (q_\infty(V_i) - q)/\tau_q(V_i) \tag{2}$$

where

$$I^g = -g_{Na}m_\infty(V_i)^3(-3(q - Bb_\infty(V_i)) + 0.85)(V_i - V_{Na}) - g_k q(V_i - V_k) - g_L(V_i - V_L)$$

$q_\infty(V_i) = n_\infty(V_i)^4 + Bb_\infty(V_i)$, $b_\infty(V_i) = (1/(1+\exp(\gamma_b(V_i + 53.3))))^4$,

$m_\infty(V_i) = a_m(V_i)/(a_m(V_i) + \beta_m(V_i))$, $n_\infty(V_i) = a_n(V_i)/(a_n(V_i) + \beta_n(V_i))$,

$\tau_q(V_i) = (\tau_b(V_i) + \tau_n(V_i))/2$, $\tau_n(V_i) = T_n/(a_n(V_i) + \beta_n(V_i))$,

$\tau_b(V_i) = T_b(1.24 + 2.678/(1+\exp((V_i + 50)/16.027)))$,

$a_n(V_i) = 0.01(V_i + 45.7)/(1 - \exp(-(V_i + 45.7)/10))$,

$a_m(V_i) = 0.1(V_i + 29.7)/(1 - \exp(-(V_i + 29.7)/10))$,

$\beta_n(V_i) = 0.125\exp(-(V_i + 55.7)/80)$, $\beta_m(V_i) = 4\exp(-(V_i + 54.7)/18)$

and

$V_{Na} = 55$ mV , $V_K = -72$ mV, $V_L = -17$ mV , $g_{Na} = 120$ mS/cm^2,

$g_K = 20$ mS/cm^2, $g_L = 0.3$ mS/cm^2 , $g_A = 47.7$ mS/cm^2,

$C = 1$ μF/cm^2, $I^b = 5$ μA/cm^2, $\gamma_b = 0.069$ mV^{-1},

$T_b = 1$ ms, $T_n = 0.52$ ms, $B = 0.21\, g_A/g_K$.

In the above equations, V_i is the voltage difference across the neuron membrane, q is a gating variable, $I^g(V_i,q)$ is the sum of the membrane currents, C is the membrane capacitance and I^b is the baseline inward current. The 3rd term on the right-hand side of eq. (1) represents neuronal coupling, where a_ℓ is the coupling parameter. $w(V_i,t)$ and $z(V_i,t)$ represent the intrinsic and extrinsic noise perturbations, respectively, while $I(V_i,t)$ represents the external stimulus current.

The general dynamical system corresponding to eq. (1) and (2) may be written as:

$$\frac{\mathrm{d}\boldsymbol{X}_i}{\mathrm{d}t} = \boldsymbol{F}(\boldsymbol{X}_i) + \boldsymbol{G}(\boldsymbol{X}_i, t), \quad i = 1, ..., N \ , \tag{3}$$

where we have defined $\boldsymbol{X}_i = [V_i, q]^{\mathrm{T}}$ as the state vector of the *i*-th oscillator, $\boldsymbol{F}(\boldsymbol{X}_i) \in \mathbb{R}^2$ as the baseline vector field and $\boldsymbol{G}(\boldsymbol{X}_i,t) \in \mathbb{R}^2$ as the cumulative effect of neuronal coupling, noise and external stimulation. The baseline system ($G \equiv 0$) admits a stable *T*-periodic limit cycle orbit, $\boldsymbol{S}(t)$.

In addition, in our case,

$$\boldsymbol{G}(\boldsymbol{X}_i, t) = \varepsilon \sum_{j=1}^{N} \boldsymbol{p}(\boldsymbol{X}_i, \boldsymbol{X}_j) + \sigma_{\mathrm{I}} \boldsymbol{H}(\boldsymbol{X}_i) \xi_i(t) + \sigma_{\mathrm{C}} \boldsymbol{Q}(\boldsymbol{X}_i) \boldsymbol{\eta}(t) + \boldsymbol{J}(\boldsymbol{X}_i, \beta) \sum_k \delta(t - \tau_k) \ , \tag{4}$$

where $\boldsymbol{p}(\boldsymbol{X}_i, \boldsymbol{X}_j) \in \mathbb{R}^2$ accounts for the effect of neuronal coupling and ε is the coupling parameter; $\xi_i(t) \in \mathbb{R}^2$ is the zero mean Gaussian white noise, added independently to each oscillator, and $\boldsymbol{\eta}(t) \in \mathbb{R}^2$ is the colored (common) noise with zero mean, unitary variance and autocorrelation function $C(t)$; $\boldsymbol{H}(\boldsymbol{X}_i) \in \mathbb{R}^{2\times2}$ and $\boldsymbol{Q}(\boldsymbol{X}_i) \in \mathbb{R}^{2\times2}$ represent the coupling of the oscillator to the independent and common noise, respectively; σ_{I} and σ_{C} denote the intensity of independent and common noise, respectively. The last term on the right-hand side of eq. (4) accounts for the effect of external stimulation. In specific, $\boldsymbol{J}(\boldsymbol{X}_i, \beta) \in \mathbb{R}^2$ represents the coupling of the oscillator to external stimulation and β represents the stimulus amplitude.

By defining the scalar phase variable $\phi_i(\boldsymbol{X}_i) \in [0,1)$, we apply the phase reduction method to eq. (3) **[2-4]**, implementing the chain rule:

$$\frac{\mathrm{d}\phi_i}{\mathrm{d}t} = \frac{\mathrm{d}\phi_i}{\mathrm{d}\boldsymbol{X}_i} \cdot \frac{\mathrm{d}\boldsymbol{X}_i}{\mathrm{d}t} = \frac{\mathrm{d}\phi_i}{\mathrm{d}\boldsymbol{X}_{\mathrm{i}}} \cdot \left(\boldsymbol{F}(\boldsymbol{X}_i) + \boldsymbol{G}(\boldsymbol{X}_i, t)\right) =$$
$$= \frac{\mathrm{d}\phi_i}{\mathrm{d}\boldsymbol{X}_{\mathrm{i}}} \cdot \left(\boldsymbol{F}(\boldsymbol{X}_i) + \varepsilon \sum_{j=1}^{N} \boldsymbol{p}(\boldsymbol{X}_i, \boldsymbol{X}_j) + \sigma_{\mathrm{I}} \boldsymbol{H}(\boldsymbol{X}_i) \xi_i(t) + \sigma_{\mathrm{C}} \boldsymbol{Q}(\boldsymbol{X}_i) \boldsymbol{\eta}(t) + \boldsymbol{J}(\boldsymbol{X}_i, \beta) \sum_k \delta(t - \tau_k) \right) \tag{5}$$

Next, we impose

$$\left. \frac{\mathrm{d}\phi_i}{\mathrm{d}\boldsymbol{X}_i} \right|_{S(\phi_i)} \cdot \boldsymbol{F}(\boldsymbol{X}_i) = \omega \, , \tag{6}$$

where $\omega\left(=1/T\right)$ is the natural frequency of the oscillator, and define the phase-sensitivity functions

$$\boldsymbol{Z}(\phi_i)=\left.\frac{\mathrm{d}\phi_i}{\mathrm{d}\boldsymbol{X}_i}\right|_{\boldsymbol{S}(\phi_i)}, \tag{7}$$

$$\boldsymbol{R}_\mathrm{I}(\phi_i)=\left.\frac{\mathrm{d}\phi_i}{\mathrm{d}\boldsymbol{X}_i}\right|_{\boldsymbol{S}(\phi_i)}\cdot\boldsymbol{H}(\boldsymbol{S}(\phi_i)), \tag{8}$$

$$\boldsymbol{R}_C(\phi_i)=\left.\frac{\mathrm{d}\phi_i}{\mathrm{d}\boldsymbol{X}_i}\right|_{\boldsymbol{S}(\phi_i)}\cdot\boldsymbol{Q}(\boldsymbol{S}(\phi_i)), \tag{9}$$

$$\varDelta(\phi_i,\beta)=\left.\frac{\mathrm{d}\phi_i}{\mathrm{d}\boldsymbol{X}_i}\right|_{\boldsymbol{S}(\phi_i)}\cdot\boldsymbol{J}(\boldsymbol{S}(\phi_i),\beta). \tag{10}$$

Assuming that the coupling strength, the noise intensities and the stimulus amplitude are small, i.e. that each oscillator remains close to the stable limit cycle, we write

$$\boldsymbol{p}(\boldsymbol{X}_i,\boldsymbol{X}_j)=\boldsymbol{p}(\boldsymbol{S}(\phi_i),\boldsymbol{S}(\phi_j))=\boldsymbol{p}(\phi_i,\phi_j). \tag{11}$$

Taking into account eq. (6)-(11), eq. (5) becomes

$$\frac{\mathrm{d}\phi_i}{\mathrm{d}t}=\omega+\varepsilon\boldsymbol{Z}(\phi_i)\sum_{j=1}^{N}\boldsymbol{p}(\phi_i,\phi_j)+\sigma_\mathrm{I}\boldsymbol{R}_\mathrm{I}(\phi_i)\boldsymbol{\xi}_i(t)+\sigma_\mathrm{C}\boldsymbol{R}_\mathrm{C}(\phi_i)\boldsymbol{\eta}(t)+\varDelta(\phi_i,\beta)\sum_k\delta(t-\tau_k) \tag{12}$$

Since perturbations affect only one degree of freedom of the dynamical system (3), namely the membrane potential V_i, eq. (12) reduces to

$$\frac{\mathrm{d}\phi_i}{\mathrm{d}t}=\omega+\varepsilon Z(\phi_i)\sum_{j=1}^{N}p(\phi_i,\phi_j)+\sigma_\mathrm{I}R_\mathrm{I}(\phi_i)\xi_i(t)+\sigma_\mathrm{C}R_\mathrm{C}(\phi_i)\eta(t)+\varDelta(\phi_i,\beta)\sum_k\delta(t-\tau_k) \tag{13}$$

Using the classical method of averaging, we may rewrite eq. (13) as **[5-7]**

$$\frac{\mathrm{d}\phi_i}{\mathrm{d}t}=\omega+a_\ell\sum_{j=1}^{N}f_\ell(\phi_j-\phi_i)+\sigma_\mathrm{I}R_\mathrm{I}(\phi_i)\xi_i(t)+\sigma_\mathrm{C}R_\mathrm{C}(\phi_i)\eta(t)+\varDelta(\phi_i,\beta)\sum_k\delta(t-\tau_k), \tag{14}$$

where $$f_\ell(\phi)=\frac{1}{2\pi}\int_0^{2\pi}Z(s)\left(V_j(\phi+s)-V_i(s)\right)\mathrm{d}s.$$

If we replace f_ℓ by its first Fourier term **[6]**, we obtain

$$\frac{\mathrm{d}\phi_i}{\mathrm{d}t}=\omega+c\sum_{j=1}^{N}\sin\left(2\pi(\phi_j-\phi_i+a)\right)+\sigma_\mathrm{I}R_\mathrm{I}(\phi_i)\xi_i(t)+\sigma_\mathrm{C}R_\mathrm{C}(\phi_i)\eta(t)+\varDelta(\phi_i,\beta)\sum_k\delta(t-\tau_k) \tag{15}$$

The coupling parameter *c* is usually considered equal to *K/N*, where *K>0* is the coupling strength. In the absence of noise perturbations and external stimulation, eq. (15) reduces to the *Sakaguchi-Kuramoto model* **[8].** Introducing the Kuramoto order parameter or the mean field defined by

$$re^{2\pi i\psi} = \frac{1}{N}\sum_{j=1}^{N} e^{2\pi i\phi_j} \ ,$$

where r characterizes the degree of synchrony and ψ is the mean phase of the oscillators, eq. (15) can be rewritten as follows

$$\frac{\mathrm{d}\phi_i}{\mathrm{d}t} = \omega + Kr\sin\left(2\pi(\psi - \phi_i + a)\right) + \sigma_{\mathrm{I}} R_{\mathrm{I}}(\phi_i)\xi_i(t) + \sigma_{\mathrm{C}} R_{\mathrm{C}}(\phi_i)\eta(t) + \Delta(\phi_i, \beta)\sum_k \delta(t - \tau_k) \qquad (16)$$

This model may be generalized to include the case of nonlinear coupling, namely a dependence of the coupling function on the mean-field amplitude r **[9]**:

$$\frac{\mathrm{d}\phi_i}{\mathrm{d}t} = \omega + Kr\sin\left(2\pi(\psi - \phi_i + a(K, r))\right) + \sigma_{\mathrm{I}} R_{\mathrm{I}}(\phi_i)\xi_i(t) + \sigma_{\mathrm{C}} R_{\mathrm{C}}(\phi_i)\eta(t) + \Delta(\phi_i, \beta)\sum_k \delta(t - \tau_k) \qquad (17)$$

Equation (17) is a Langevin equation which may be interpreted in the Stratonovich sense. It is convenient to convert this equation into a white-noise Langevin equation **[10]**:

$$\frac{\mathrm{d}\phi_i}{\mathrm{d}t} = \omega + Kr\sin\left(2\pi(\psi - \phi_i + a(K, r))\right) + v_{\mathrm{C}} + \left(\sigma_{\mathrm{I}} R_{\mathrm{I}}(\phi_i) + \sqrt{D_{\mathrm{C}}}\right)\xi_i(t) + \Delta(\phi_i, \beta)\sum_k \delta(t - \tau_k), \qquad (18)$$

where $v_C \approx \sigma_{\mathrm{C}}^2 \int_0^\infty ds\, C(s) \int_0^1 d\phi_i\, R'_{\mathrm{C}}(\phi_i) R_{\mathrm{C}}(\phi_i - \omega s)$ and $D_{\mathrm{C}} \approx \sigma_{\mathrm{C}}^2 \int_{-\infty}^\infty ds\, C(s) \int_0^1 d\phi_i\, R_{\mathrm{C}}(\phi_i) R_{\mathrm{C}}(\phi_i - \omega s)$

represent the effective drift and diffusion coefficient, respectively.

The Stratonovich eq. (18) may be converted into an equivalent Ito stochastic differential equation **[11]**:

$$\frac{\mathrm{d}\phi_i}{\mathrm{d}t} = \omega + Kr\sin\left(2\pi(\psi - \phi_i + a(K, r))\right) + v_{\mathrm{C}} + \left(\sigma_{\mathrm{I}} R_{\mathrm{I}}(\phi_i) + \sqrt{D_{\mathrm{C}}}\right)\xi_i(t) + \frac{\sigma_{\mathrm{I}}}{2} R'_{\mathrm{I}}(\phi_i)\left(\sigma_{\mathrm{I}} R_{\mathrm{I}}(\phi_i) + \sqrt{D_{\mathrm{C}}}\right) + \Delta(\phi_i, \beta)\sum_k \delta(t - \tau_k) \qquad (19)$$

1. Rose RM, Hindmarsh JL. The assembly of ionic currents in a thalamic neuron I. The three-dimensional model. Proceedings of the Royal Society of London B: Biological Sciences.1989; 237: 267-88.

2. Kuramoto Y. Chemical Oscillations, Waves, and Turbulence: Springer New York; 1984.

3. Galán RF. The phase oscillator approximation in neuroscience: an analytical framework to study coherent activity in neural networks. In: Coordinated Activity in the Brain: Springer New York; 2009. p. 65-89.

4. Brown EN, Moehlis J, and Holmes P. On the phase reduction and response dynamics of neural oscillator populations. Neural computation.2004; 16(4): 673-715.

5. Kori H, Kawamura Y, Nakao H, Arai K, Kuramoto Y. Collective-phase description of coupled oscillators with general network structure. Phys. Rev. E. 2009; 80(3): 036207.

6. Izhikevich EM. Dynamical systems in neuroscience: MIT press; 2007.

7. Nabi A, Moehlis J. Single input optimal control for globally coupled neuron networks. J. Neural Eng. 2011; 8(6): 065008.

8. Sakaguchi H, Kuramoto Y. A soluble active rotator model showing phase transitions via mutual entertainment. Progress of Theoretical Physics. 1986; 76(3): 576-81.

9. Baibolatov Y, Rosenblum M, Zhanabaev ZZ, Kyzgarina M, Pikovsky A. Periodically forced ensemble of nonlinearly coupled oscillators: From partial to full synchrony. Physical Review E. 2009; 80(4): 046211.

10. Nakao H, Teramae JN, Goldobin DS, Kuramoto Y. Effective long-time phase dynamics of limit-cycle oscillators driven by weak colored noise. Chaos. 2010; 20(3): 033126.

11. Gardiner C. Handbook of Stochastic Methods: Springer Berlin; 1985.
